# 45 years of directional wave recorded data at the Acqua Alta oceanographic tower

**DOI:** 10.1038/s41597-025-04541-8

**Published:** 2025-02-06

**Authors:** Luigi Cavaleri, Angela Pomaro, Luciana Bertotti, Andrea Bonometto, Luca De Nat, Alvise Papa, Valerio Volpe

**Affiliations:** 1 National Research Council of Italy, Institute of Marine Sciences, Venice, Italy; 2 Sapienza University of Rome, Department of Computer, Control, and Management Engineering Antonio Ruberti, Rome, Italy; 3 Istituto Superiore per la Protezione e la Ricerca Ambientale, Venice, Italy; 4 Consorzio Venezia Nuova, Venice, Italy; 5 Centro Previsioni e Segnalazioni Maree, Municipality of Venice, Venice, Italy; 6 Provveditorato Interregionale per le Opere Pubbliche per il Veneto, Trentino Alto Adige e Friuli Venezia Giulia, Venice, Italy

**Keywords:** Physical oceanography, Physical oceanography

## Abstract

The dataset comprises a 45-year-long directional wave time series recorded at the Acqua Alta Oceanographic research Tower (AAOT) since 1979. The AAOT is located in the Northern Adriatic Sea and it is managed by the Institute of Marine Sciences of the National Research Council of Italy (CNR-ISMAR). The extent of the time series enables the description of the wave climate in the North Adriatic region and the identification of trends and links with large-scale climate patterns from a single and permanent observational source. Different wave gauges have been used since the start of the measurements, progressively upgraded and repositioned during maintenance operations. The recent addition of more precise instruments and the availability of the related raw dataset allowed for a re-evaluation of the previously published data, while extending the timeseries in time. This has enabled the creation of a substantially improved, yet homogeneous, measured dataset, thereby enhancing the reliability of the related long-term scenario analysis.

## Background & Summary

**Fig. 1:**
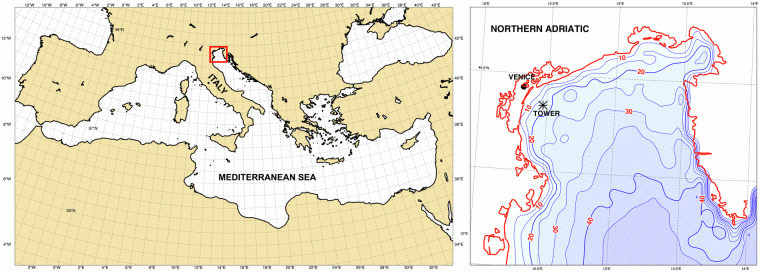
Identification of the area of interest. Left panel: the Northern Adriatic basin, East of Italy, within the Mediterranean Sea, as part of the European region. Right panel (enlargement of the small rectangle): the Northern Adriatic basin, the Venice Lagoon and the Acqua Alta Oceanographic Tower position located about 15 km off the coastline.

**Fig. 2:**
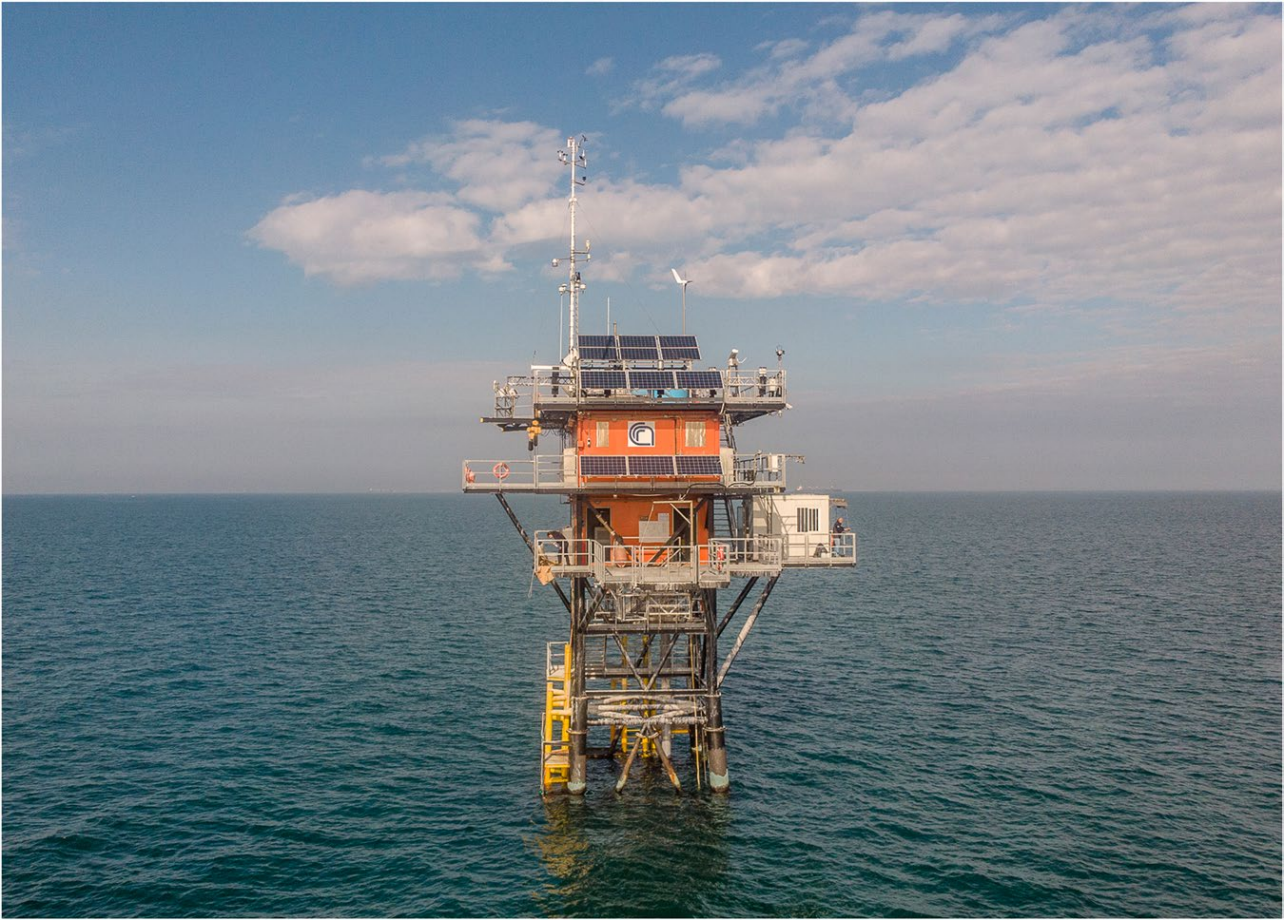
The Acqua Alta Oceanographic Tower. Photo by Marco Zorzanello.

The area of interest is the northern part of the Adriatic Sea, the elongated basin to the East of Italy, as illustrated in Fig. 1. The basin is approximately 700 km long and 200 km wide. Following the disastrous events of 4 November 1966 (a comprehensive description of the storm and its consequences, particularly in Venice, can be found in the literature^1^), a dedicated scientific research institute was established and the Acqua Alta oceanographic tower was set into position soon after. Figure 2 depicts the research facility, fully described in^2^. Relevant for the later discussion, we point out that the four legs of the basic square-framed structure are aligned with the four cardinal directions.

While detailed wave measurements were taken during specific storms^3^, long-term regular measurements were initiated in 1979^4^. Located 15 km from the coastline, facing the inlets to the Venice lagoon, GPS coordinates 45° 18′ 51.288i″ N, 12° 30′ 29.694″ E, the tower has been a valuable source of offshore meteorological and oceanographic data on the local marine environment. Indeed, at present it is difficult to realize how little was known at the time about what weather and sea were and could do. Some high-level, officially qualified information about the sea may appear naive today (e.g., “waves cannot be higher than 3 m in the Adriatic Sea”), but it is representative of the knowledge and data availability at the time.

With the due changes of instruments and recording apparatus, wave data were continuously collected and disseminated as a 39-year-long homogeneous time series^5,6^. In the subsequent years, while measurements were continued over time, new more accurate and complete recording systems were added. In addition to extending the timeseries, this led to an *a posteriori* additional revision and correction of part of the data. The objective of this paper is to describe the aforementioned non-trivial corrections, and to further extend the whole time series until May 2024, for an overall extent of 45 years. The updated dataset provides data at 15-minute intervals extending the information previously made available on a three-hourly basis.

In the Methods section we describe the data collected since 1979, the measuring instruments employed, and the inherent advantages and limitations of the various solutions used over time. With respect to the 2018 publication^5,6^, this paper describes the further corrections implemented for the 2004–2018 period and the new data available until May 2024. Three complementary time series of wave data are provided, respectively: a) the spectral, or variance-derived, significant wave height Hm0; b) the corresponding H1/3, i.e., the mean of the highest 1/3 wave heights in a record; and c) the model-derived time series. An illustrative example is provided by 22 December 1979, when part of the on-board structures was destroyed by unexpected (at the time) high waves. Consequently, no record is available. Furthermore, model data for the historical storm of 4 November 1966 have been incorporated.

Focusing on the specific area, the Northern Adriatic Sea, situated at the upper end of this mostly enclosed basin, may not be considered fully representative of the broader wave climate of the Mediterranean Sea. However, its geographical position, to the south of the Eastern Alps and basically open to south, yet lacking a substantial wave energy background from distant sources, makes it extremely sensitive to even minor alterations in the prevailing meteorological conditions. Two distinct wave conditions dominate the local area: Sirocco, blowing from south-east, is associated with a typical large-scale westerly flow, with possible Mediterranean cyclogeneses; Bora, on the other hand, is associated with the cold easterly flow that is typical of a high-pressure system entering Europe from the west. A mixed situation with a low-pressure centre located West of Italy is also possible (Bora Scura, i.e., Dark Bora, because often associated to rain). A multidecadal wave time series, as illustrated here, can clearly demonstrate the long-term trend of these two situations and how climate, i.e., the dominant regime, is changing over time.

As an integrated effect, in space and time, of the local meteorological situations, long-term wave data are extremely useful for summarizing atmospheric information in a compact form^7^, given the sparser nature of such data. Extended meteorological reanalyses, notably the fifth generation ECMWF reanalysis ERA5^8,9^, provide data spanning several decades (1940 to the present). However, their relatively coarse resolution (31 km for ERA5) may dampen and smooth the relevant signal in challenging areas. Conversely, these are frequently sensitive to even minor alterations in the prevailing situation, as it is evident in the present case. Consequently, the extensive local records over extended periods of time are of considerable value.

## Methods

This section provides a detailed description of the measuring instruments used to collect the aforementioned data. From 1979 onwards, the recording equipment underwent a series of technological advancements. This is clearly reflected in the data that has been collected and the information that has been derived from it. The potentially implied lack of homogeneity has already been discussed in^5^, but it is here briefly reported for a complete information. Five distinct data sections are identified and individually described with all the required characteristics: 1979–1987, 1987–2004, 2004–2014, 2014–2017, and 2017-present.

### First period 1979–1987

The first directional wave recording system was installed on the Acqua Alta Oceanographic Tower (AAOT) in early 1979. The fundamental instrumentation comprised two compact borehole pressure transducers, manufactured by Bell & Howell and equipped with vented gauges, which were installed at an average depth of 4.0 metres. The instrument’s key advantages were its relatively low cost, durability, accuracy and minimal power consumption. The two instruments were powered by the onboard batteries at three-hour intervals, with a recording period of 10 minutes at a 2 Hz frequency. A higher frequency would have been of no use due to the attenuation of waves with depth. The transducers were located on two opposing legs of the tower, north and south. A pulley system was employed to place the instruments at their required depth and recover them for periodic maintenance, with bio-fouling representing the most significant issue. In accordance with linear theory^10^ and for the local 16-metre depth, information was only available for 2.5-second waves or longer.

The transducers were powered at three-hour intervals, in accordance with the synoptic timescale. Once the system was operational, the initial minute of each record was evaluated to ascertain whether the prevailing sea conditions were deemed worthy of further observation. In the event that the conditions were not considered suitable, the data was not included in the final analysis. In the event of conditions deemed interesting, a full ten-minute record was taken and stored on a computer-compatible Kennedy tape recorder (model 1600, 7 tracks, 200 bpi, data rate 500 ch./s). The time lag between the scans of the two transducers was 0.01 seconds.

The operational range of the pressure transducers was 0–25 psi, with a full deflection of 5 VDC. Given the local depth, hence the practical limit of the local maximum wave height, a reduced range 0–3 VDC range was used. This range corresponded to a 12.4 m water column. Given the potential for local surge of up to 2 m, it is notable that more than 6 m crest heights were still recorded. The resolution of the recording system was 1/1000, equivalent to 1.24 cm at the pressure transducer level.

In the subsequent data analysis phase, each record was first transformed into water height using appropriate calibration factors. The average value (in practice, the transducer depth) was then subtracted (the maximum tidal variation in 10 minutes equals approximately 3 cm). If the event that the derived surface displacement was less than 20 cm, the record was excluded. In the alternative, the two series were analyzed via the Fast Fourier Transform (FFT) technique. The routine analysis comprised the evaluation of the spectrum and cross-spectrum, which were then used to derive the related mean direction. Taking into consideration the attenuation of waves with depth, integrated parameters, including significant wave height (Hm0), wave mean and peak periods and mean directions were derived. About direction, it should be noted that, in principle, two measurement points would result in a specular ambiguity. However, the local basin geometry ensures that all the relevant waves originate from the eastern quadrants.

### Second period 1987–2004

A limitation to the use of only two pressure transducers is the lack of information on directional spreading for each spectral frequency range. Given the location of the tower, this is not a strictly necessary consideration, as the northeast short fetch and the much longer southeast one lead to different wavelengths, which in turn give rise to different frequencies of interest in the actual records. It is possible that some degree of superposition may still occur in certain conditions. Therefore, the deployment of a third transducer at the previously used depth along the eastern leg of the tower was a valuable strategy.

Other relevant modifications included the use of absolute pressure transducers, which exhibited reduced sensitivity to malfunctioning and venting pipe obstruction. The effect of tidal variations in depth was evaluated using data from the on-board fast-response tide gauge^11^.

The resolution of the recording system was 1/256, which introduced one unit of white noise into the analysis, manifesting as a white spectrum. Negligible in itself in the original signal, this noise was markedly amplified in the high-frequency range of the spectrum (due to the correspondingly large attenuation with depth). This indicated that a convenient cut-off point could be identified, after which the spectra were fitted with an f^−5^ frequency tail for the purposes of energy estimation.

The procedure employed for the directional analysis is thoroughly documented in the existing literature^10^. For a concise overview, please refer to the previously published manuscript on this timeseries^5^.

### Third period 2004–2014

While pressure transducers offer numerous advantages, they are particularly susceptible to maintenance issues, especially in a highly dynamic environment such as the Northern Adriatic Sea, characterized by also a strong fouling. Since 2004 the official recording system has been an echo-sounder, located on the eastern corner of the tower, 1.5 m from the structure and at the second deck level of the AAOT. Given that the measurements were now taken from above the surface, it was of interest to consider also the tail of the spectrum. Hence a 4 Hz sampling frequency was used.

Given that the air sound speed is the critical quantity for estimating the distance from the sea surface, the air temperature was accounted for using a shielded (from solar radiation) temperature transducer.

Over time, the recording periods have varied. Until 4 September 2013, records were made for a period of three minutes. Subsequently, records of five minutes’ duration were made for a period of 16 days, after which each record was extended to 15 minutes. All data is available at 15-minute intervals. The raw data was not retained; instead, it was erased following the zero-crossing analysis. This yielded the values for H1/3, Hmax, Tm, and Tmax. These measurements are ongoing.

With respect to the 2018 published time series^5^, substantial corrections have been made to this data section, based on the additional sea state measurements available at the AAOT. This has enabled inter-calibration and subsequent reprocessing of the previously available dataset. The methodology employed is fully documented and explained in the Technical Validation section.

### Fourth Period 2014–2017

In 2014, a novel wave gauge was installed on the tower: a down-looking radar whose 2 Hz signal continuously explores the distance from the surface. The most recent 15 minutes of continuous data (1800 data points) are analysed at five-minute intervals, with the results stored on board and also transmitted to the mainland. Following their use, the raw data was cancelled. In the present study, the long-term on-board time series data, collected at 15-minute intervals, was considered as a follow-up to the previous period. Each set of 1800 points set is analysed using the zero-crossing technique, which provides the corresponding H1/3 and corresponding mean wave period (Tm) values.

### Fifth period 2017-present

It is notable that between June and October 2017, the tower underwent significant renovation works. Indeed, the structure was installed in March 1970 and, due to prolonged exposure to the elements, had become visibly degraded after approximately half a century. In practice, the template, i.e., the submerged, bottom-implanted and four metres emerging structure, was maintained. This was done while pre-constructing and substituting the working and living upper section. This resulted in a period of data absence, after which the same wave-measuring instruments were reinstalled in October 2017. The primary improvement was the implementation of a system for the regular storage and periodic retrieval of the 2 Hz data from the radar wave measuring system. This has allowed the direct evaluation of both H1/3 and the spectrally derived Hm0 from the same raw data, as well as the usual frequency (hence period) mean and peak quantities, which are documented in the following paragraph. Furthermore, a second independent radar system, with a similar configuration, was deployed in 2017, but with only H1/3 and the corresponding frequency parameters recorded. However, the contemporary availability of three instruments, including the one with full 2 Hz time-series raw data, has permitted a rigorous intercomparison, validation and consequent calibration, which have also prompted the aforementioned reprocessing of part of the previously available measurements.

## Data Records

The dataset is available at PANGAEA.974074^12^ with this section being the primary source of information on the availability and content of the data being described. PANGAEA is operated as an open access library for archiving, publishing, and distributing georeferenced data from earth and environmental sciences. It focuses on observational and experimental data^13^.

Starting in 1979, this extended dataset comprises a time series of the conventional integral parameters, namely significant wave height Hm0 (not H1/3), mean frequency and period fm and Tm, peak frequency and period fp and Tp, and the overall mean wave direction. The latter information has been provided since 2004 by the local wave modelling system Henetus. This model has been operational for the Adriatic Sea since 1991 and has been developed and operated by CNR-ISMAR. It has been continuously upgraded, particularly through the correction of the ECMWF wind fields input. Extensive validation has been provided and is available from the literature^14^.

The updated dataset provides data at 15-minute intervals, where measurements are available, i.e., since 2004, extending the information previously made available on a three-hourly basis. We note the availability of directional data together with Hm0 and Tm parameters. According to our experience, this provides relevant insights into the impact of climate change over the entire Mediterranean Region, from the unique perspective of a long-term timeseries of observational data in a particularly sensitive area.

As anticipated in the initial Background & Summary, three different time series are provided. The initial series reports the Hm0 values, as measured up until 2004, and subsequently derived from the available H1/3 data. Conversely, the second time series presents the H1/3 time series, derived from Hm0 until 2004 and subsequently measured using the available systems. These two time series presented here reflect only data obtained from measurements. As previously indicated, in certain instances, particularly during the early years, the datasets lacked records for particularly intense storms. While not strictly relevant for the general distribution, this missing data becomes crucial when working on extremes statistics. In particular, the storm that occurred on 22 December 1979 is considered, derived by model hindcast^1^, similar as severity to the fully documented event of 29 October 2018, with up to 6 m Hm0 measured at the AAOT. Furthermore, the Hm0 time series is integrated with model data, where available. While not continuous, this data is relevant for extreme events, as evidenced by the addition of wave model data for the 4 November 1966 event. This storm, the most severe in recent memory, is included due to its notable impact on both the physical environment and human society. As previously discussed in the Background & Summary, the damages caused by this storm, along with the disastrous flooding of Venice, captured global attention, leading to the establishment of the CNR-Institute of Marine Sciences and the installation of the AAOT, where all the data presented in this study was collected.

## Technical Validation

In consideration of the published time series^5^, two fundamental corrections have been implemented.

The recent availability of the radar data, which has been fully superimposed on the acoustic data, has allowed a direct comparison and calibration of the respective data and time series. This comparison also permitted the realisation that the echo-sounder data available for the period 2004–2017, which was acquired and reported as significant wave height, was in fact representative of H1/3. The discrepancy between Hm0, i.e., the integral of a measured or modelled wave spectrum, and H1/3 is typically between 6% and 8%. This depends on the local environment^15^. In most cases, Hm0 is greater than H1/3. The two corrections are outlined in the following sections.

**Fig. 3:**
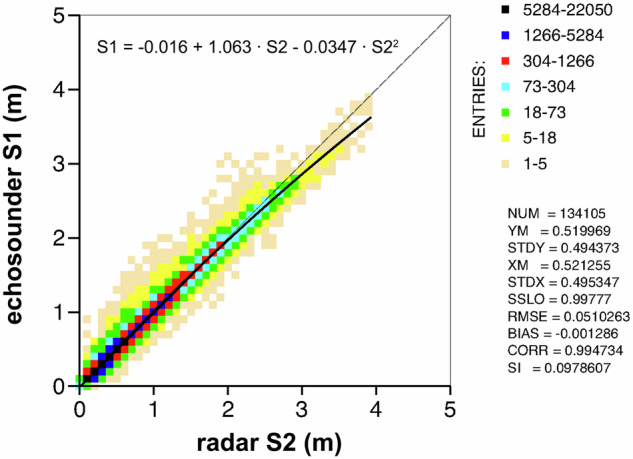
Scatter diagram between S1 (echosounder) and S2 (radar system where also raw data is available) measurements. Both the related linear and second order best-fits are shown. The numbers on the right refer to the linear fit, the upper expression is the quadratic one.

For the sake of simplicity, we will refer to the echosounder data available since 2004 as System 1 (S1); the first system radar gauge data as System 2 (S2), and the more recent second system radar gauge data as System 3 (S3). Prior research^16^ indicates that the radar data is of superior quality with respect to the echosounder one. In any case, Fig. 3 presents the scatter diagram between S1 and S2 without any assumption. These findings are also supported by a comparison with the local wave analysis and forecast system^14^. Similarly, a consistent analysis of the related integrated parameters, namely wave period and Hm0/Tm distributions, has been performed. It can be concluded that, for the purposes of this study, the third period (2004–2014) described in the Methods section requires a calibration corresponding to the square regression curve, whose analytical best-fit expression is reported in the aforementioned figure. This is used to derive, for each S1, the corresponding corrected value.

Subsequently we encounter the dichotomy Hm0, H1/3. As already mentioned^12^, the discrepancy between these values is typically 6–8% (Hm0 > H1/3). However, the specific, more common value is subject to variation in accordance with the typical local conditions (e.g., the presence of more or less active and strong wind sea or swell) prevailing in the area under consideration. In the area of interest, this was investigated using the 2 Hz data from source S2. Granted some limited scattering associated to the random nature of the process, a ratio of 1.082 was obtained. For practical purposes, this ratio was set to 1.08.

Additionally, the third period S2 analysis, for which the raw data is available, was done with down-zero crossing. It is unclear whether the echosounder data analysis was done with up- or down-zero-crossing, but the differences would be random and minimal, in any case included in the calibration presented in Fig. 3.

To ensure the completeness of the time series, directional information from 2004 onwards has been provided (mean flux direction) using the Henetus wave model data^14^.

In the aforementioned previous publication^5^, the statistics of the H-T distribution were employed to elucidate an issue pertaining to the depth of the pressure transducers in the 1987–2004 segment of the data, which was subsequently fully resolved. The consistency of the five periods under consideration is verified by checking their singular H-T distributions. Within the expected natural variability, the five distributions are found to be consistent with each other, confirming the uniform quality of the data.

## Data Availability

Data cleaning and analysis associated with the current submission have been performed in Fortran code with standard and commonly used procedures.
